# The Fagerström and AUDIT Tests as Probable Screening Tools in Oral Cancer and Their Correlation with *CYP1A1*, *GSTM1*, *GSTP1*, and *GSTT1* Gene Expression

**DOI:** 10.3390/ijerph19073991

**Published:** 2022-03-27

**Authors:** Celso Muller Bandeira, Adriana Ávila Almeida, Mônica Ghislaine Oliveira Alves, Maria Beatriz Nogueira Pascoal, José Francisco Sales Chagas, Morun Bernardino Neto, Patrícia Pimentel de Barros, Fábio Daumas Nunes, Celina Faig Lima Carta, Janete Dias Almeida

**Affiliations:** 1 Department of Bioscience and Oral Diagnosis, Institute of Science and Technology of São José dos Campos, São Paulo State University (Unesp), São José dos Campos 12245-000, Brazil; celso.bandeira@humanitas.edu.br (C.M.B.); adrianaavilaalmeida@yahoo.com.br (A.Á.A.); barrosdnapp@yahoo.com.br (P.P.d.B.); celinafaig@yahoo.com.br (C.F.L.C.); 2 Faculdade de Ciências Médicas de São José dos Campos—Humanitas, São José dos Campos 12220-061, Brazil; 3 School of Medicine, Anhembi Morumbi University, São José dos Campos 12230-002, Brazil; mgoliveiraalves@gmail.com; 4 Technology Research Center (NPT), Universidade Mogi das Cruzes, Mogi das Cruzes 08780-911, Brazil; 5 Department of Head and Neck Surgery, São Leopoldo Mandic College, Campinas 13045-755, Brazil; maribibia@ig.com.br (M.B.N.P.); josechagas@ig.com.br (J.F.S.C.); 6 Department of Head and Neck Surgery, Hospital Municipal Doutor Mário Gatti, Campinas 13036-902, Brazil; 7 Department of Basic Sciences and Environment, São Paulo University, São Paulo 12602-810, Brazil; morun@usp.br; 8 Department of Oral Pathology, School of Dentistry, São Paulo University, São Paulo 05508-000, Brazil; fadnunes@usp.br

**Keywords:** buccal mucosa, carcinogenesis, gene expression, mouth neoplasms, squamous cell carcinoma, xenobiotic

## Abstract

Background: Cancer is currently a major public health problem worldwide, with a marked increase of about 70% in the number of expected diagnosed cases over the next two decades. The amount of tobacco and alcohol consumed is calculated based on the subjective information provided by the user. Tobacco exposure can be assessed using the Fagerström Test for Cigarette Dependence (FTCD) and alcohol consumption by the Alcohol Use Disorder Identification Test (AUDIT). Materials and Methods: Forty-eight subjects answered the Fagerström, and AUDIT tests and we studied them as likely screening tools for oral cancer and their correlation with the expression of *CYP1A1*, *GSTM1*, *GSTP1*, and *GSTT1* genes by the RT-qPCR method. Results: There were significant differences in the AUDIT score and *CYP1A1* expression between cancer and control groups. Participants in advanced stages, whether due to tumor size or regional metastasis, showed significant differences in the duration of tobacco use, FTCD, AUDIT score, and *CYP1A1* expression when compared to patients in early stages. Among subjects without cancer, we found a significant correlation between participant age and *GSTP1* expression. Furthermore, the expression of *GSTP1* was significantly correlated with the number of cigarettes smoked per day, duration of tobacco use, and FTCD. Conclusions: Questionnaires designed to evaluate the degree of tobacco and alcohol exposure and dependence combined with gene expression tests can be useful to assess the risk of developing oral cancer. Furthermore, raising the awareness of individuals regarding their degree of dependence and encouraging them to participate in cessation programs are important educational measures for the prevention of tobacco-related malignancies.

## 1. Introduction

Cancer is currently the second largest public health problem worldwide, with a marked increase of about 70% in the number of diagnosed cases being expected over the next two decades [1]. In developed countries, cancer is related to higher sociocultural status and longevity, although its main consequences in terms of morbidity and mortality are found in low-income populations, especially in developing countries [1]. About 20 new cases of oral cancer per 100,000 inhabitants are diagnosed in Brazil every year [1,2,3]. Squamous cell carcinoma (SCC) is the most common malignant neoplasm of the head and neck region. The diagnosis, treatment, and prognosis of head and neck tumors are based on the TNM classification (UICC/AJCC), which has been updated by the inclusion of molecular factors [4,5].

It is estimated that about 1.5 billion people worldwide use tobacco products [1,6]. Tobacco use is classified as an addictive disease by the World Health Organization (WHO) and is the main cause of cancer in the world [7]. Like tobacco, alcohol consumption is an important risk factor for head and neck cancers, which participates not only in the carcinogenic process but also acts as a potentially addictive drug [8,9,10]. Smoking, associated with excessive consumption of alcoholic beverages, increases by up to 15 times the risk of oral cancer [10,11,12,13,14,15].

*CYP1A1* is one of the most important genes responsible for the bioactivation of tobacco carcinogenic substances including polycyclic aromatic hydrocarbons (PAH) and nitrosamines and benzopyrenes, as well as alcohol metabolism [16]. Phase II of glutathione S-transferase (GSTs) enzymes has an important role in the detoxification of carcinogens, and especially the polycyclic aromatic hydrocarbons (PAH) found in tobacco smoke [17,18,19].

Over the past decades, many highly toxic combustion compounds, known as carcinogens, have been identified in tobacco-related products. These compounds include benzopyrenes and other polycyclic aromatic hydrocarbons derived from nicotine, such as nicotine-specific nitrosamines. Other toxic substances such as benzene, formaldehyde, carbon monoxide, cyanide, acrolein, and polonium can also be found [20]. These toxic compounds are activated and detoxified in two phases. In Phase I, the carcinogens are metabolized by cytochrome P450 enzymes to become more soluble for excretion or more electrophilic for elimination by Phase II glutathione S-transferase (GSTs) enzymes [21]. These enzymes are highly polymorphic, and their prevalence varies among different populations; however, in Brazil, the prevalence of these polymorphisms is low because of the extensive miscegenation among the three main ethnic colonization groups [22,23,24,25].

The amount of tobacco and alcohol consumed is usually calculated by subjective inference from the information provided by the user. The pack-years variable is widely used to estimate tobacco consumption during the smoker’s life, but the degree of dependence on cigarettes which can estimate the life exposure of tobacco may be assessed using the Fagerström Test for Cigarette Dependence (FTCD) [26]. Alcohol consumption depends on variables such as the type and content of alcohol in each product and the frequency and intensity of use, as well as the real magnitude of intake described by the user. The Alcohol Use Disorder Identification Test (AUDIT) was developed by the WHO as a simple and validated tool for alcohol use screening [27].

Since oral cancer is a global health concern and alcohol and tobacco consumption are major risk factors involved in its carcinogenesis, the aim of this study was to evaluate the Fagerström and AUDIT tests as likely screening tools for oral cancer and their correlation with the expression of *CYP1A1*, *GSTM1*, *GSTP1*, and *GSTT1* genes measured by the RT-qPCR method. Questionnaires to assess the degree of exposure and dependence, such as tobacco and alcohol associated with simple genotyping tests, can be a useful tool to assess the risks of developing oral cancer.

## 2. Materials and Methods

### 2.1. Sample Selection

This is an observational study with a cross-sectional and ecological design. The project was submitted and approved by the Research Ethics Committee (CAAE No. 42387315.0.0000.0077) and was conducted in accordance with the ethical principles of the Declaration of Helsinki. All participants received information about the aims of the study and agreed to participate by signing the informed consent form. Samples were collected from patients over 18 years of age, smokers or non-smokers, with a diagnosis of oral SCC, who sequentially attended the Celso Pierro Hospital of the Pontifical Catholic University of Campinas/SP (PUC-Campinas) and the Mario Gatti Municipal Hospital, Campinas/SP. As the control group, tissue fragments were collected from the healthy border at least 1 cm away from a benign lesion such as mucocele and fibroma of patients treated at the Oral Diagnosis Clinic, Department of Biosciences and Oral Diagnosis, Institute of Science and Technology/Unesp, and at the Municipal Health Department in São Sebastião/SP. The samples were collected during incisional biopsy or surgical treatment and sent for anatomopathological examination. Patients with a history of and treated for malignant neoplasms in any other organ or system or patients with lip cancer were not included, as it is related to sun exposure and not tobacco and alcohol. Information on TNM staging was collected for cancer patients [28].

### 2.2. Evaluation of Tobacco and Alcohol Dependence

Nicotine dependence was evaluated using the FTCD [26,29]. This questionnaire consists of six questions and is scored according to the answers. In the end, the patient’s nicotine dependence is classified into five categories: very low (0 to 2 points), low (3 to 4 points), moderate (5 points), high (6 to 7 points), and very high (8 to 10 points). The cut-off for nicotine dependence was ≥4 points [30]. The AUDIT was used to estimate risk behavior related to alcohol consumption. This instrument consists of 10 questions that identify four patterns of alcohol consumption: low risk use (0 to 7 points), risk use (8 to 15 points), harmful use (16 to 19 points), and probable dependence (≥20 points). The cut-off for risk consumption was 8 points [27].

### 2.3. RNA Extraction and Quantification

All samples were stored in an Eppendorf tube with Allprotect tissue reagent (Qiagen, Carlsbad, CA, USA) overnight at 4 °C and then at −80 °C until use. Total RNA was extracted using the TRIzol kit (Ambion, Inc., Carlsbad, CA, USA) according to manufacturer recommendations. TRIzol (1 mL) was added to a 2.0 mL microtube containing solid tumor tissue and the mixture was incubated at room temperature for 10 min. Next, 200 μL chloroform (Sigma-Aldrich, St. Louis, MO, USA) was added and the microtubes were centrifuged at 12,000× *g* for 15 min at 4 °C. The supernatant was transferred to a new tube with 500 µL isopropanol (Sigma-Aldrich, St. Louis, MO, USA). After centrifugation, the pellet was washed with 70% ethanol (Sigma-Aldrich, St. Louis, MO, USA), centrifuged again, and resuspended in 50 µL of RNA storage buffer (Ambion, Inc., Carlsbad, CA, USA). The concentration, purity, and quality of the RNA were verified in a NanoDrop 2000 spectrophotometer (Thermo Fisher Scientific, Inc., Wilmington, DE, USA) and RNA was visualized under a transilluminator. The total RNA extracted (1 µg) was treated with DNase I (Turbo DNase Treatment and Removal Reagents, Ambion, Inc., Carlsbad, CA, USA) and transcribed into complementary DNA (cDNA) using the SuperScript^®^ III First-Strand Synthesis SuperMix for RT-qPCR kit (Invitrogen^TM^, Carlsbad, CA, USA) according to the protocols recommended by the manufacturer.

### 2.4. Selection of the Genes Studied

Three reference genes were selected: *ACTB*, *GAPDH*, and *TUBA1C*. The last was selected because it showed the highest compatibility with the samples. The primer sequences were confirmed on the NCBI/GenBank website, which was specific for the Homo sapiens species and homology. The following primers were chosen for the study: *CYP1A1* (Fwd.—CTTCCGACACTCTTCCTTCG, Rev.—GGTTGATCTGCCACTGGTTT); *GSTM1* (Fwd.—ACTTGATTGATGGGGCTCAC, Rev.—ATGGTTGTCCATGGTCTGGT); *GSTP1* (Fwd.—CAGGTGTCAGGTGAGCTCTG, Rev.—ATGACCCGTTACTTGGCTGG); *GSTT1* (Fwd.—GTTGCTCGAGGACAAGTTCC, Rev.—ATCAGCTCCGTGATGGCTAC); *TUBA1C* (Fwd.—CCGGGCAGTGTTTGTAGACT, Rev.—TTGCCTGTGATGAGTTGCTC). The efficiency of the primers was tested, and the amount and concentration of cDNA and annealing temperature were standardized. All primers showed efficiency between 95 and 154%.

### 2.5. Gene Expression Analysis

The RT-qPCR method was applied to evaluate the amount of cDNA in the exponential phase of the amplification reaction. The SYBR^®^ Green fluorophore (Platinum^®^ SYBR^®^ Green RT-qPCR SuperMix-UDG, Applied Biosystems, Framingham, MA, USA) was used in the following reaction mixture: 12.5 µL of Platinum SYBR Green SuperMix, 1 μL of ROX (reference dye), 300 µM of the forward primer, 300 µM of the reverse primer, 2 μL of cDNA solution, and 2.1 µL of Ultrapure water (Invitrogen^TM^, Carlsbad, CA, USA) to obtain a final volume of 20 µL in each well of a 96-well plate (Invitrogen^TM^, Carlsbad, CA, USA). The wells were sealed with an optical adhesive film (Invitrogen^TM^, Carlsbad, CA, USA) and the plates were placed in a StepOnePlus™ System (Applied Biosystems, Framingham, MA, USA). The following cycling parameters were used: 50 °C for 2 min, followed by initial denaturation at 95 °C for 2 min and 40 cycles at 95 °C for 15 s and 60 °C for 30 s. After the last cycle, the samples were subjected to dissociation (melting) curve analysis at intervals of 0.1 °C. No bimodal curve or abnormal amplification signal was observed. The 2^−ΔΔCT^ method was used to determine the relative changes in gene expression [31].

### 2.6. Statistical Analysis

The sample was analyzed regarding gender, smoking status (smoker and non-smoker), tobacco consumed in cigarettes per day, and duration of tobacco consumption. The consumption pattern of alcoholic beverages was also evaluated. Tumors were divided into early (stages I and II) and advanced stages (stages III and IV). The normality of the data was evaluated by the D’Agostino–Pearson test. Non-parametric data are reported as the median and interquartile range (IQR). The groups were compared by the Mann–Whitney test. Spearman’s correlation coefficient was used to evaluate the correlation between the variables studied. A significance level of 5% was adopted for all tests. The GraphPad Prisma software was used for data analysis.

## 3. Results

**Figure 1:**
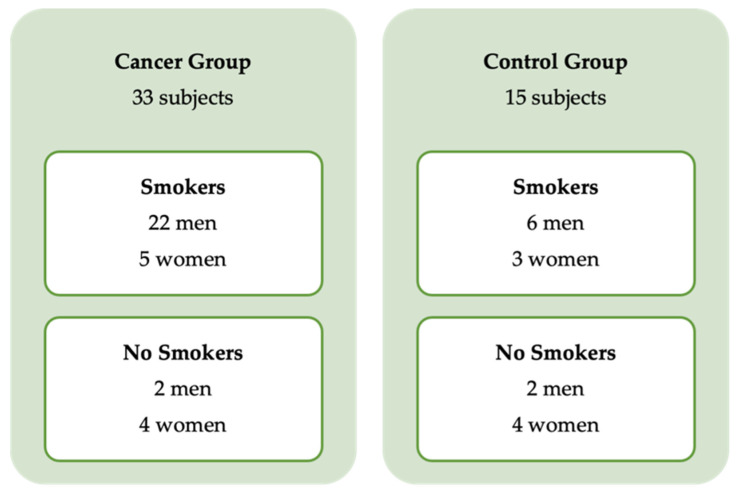
Sample diagram.

Forty-eight subjects, 32 men (67%) and 16 women (33%), with a mean age of 55 ± 14.5 years, were included. Thirty-three participants had cancer (24 men and 9 women) and twenty-seven of them were smokers (22 men and 5 women) (Figure 1). The AUDIT results in the cancer group presented statistical significance between the genders (*p* = 0.035) with 19 men and 2 women classified as behavioral risk score (≥8 points) (*p* = 0.002). The remaining 15 subjects with benign lesions included 8 men and 7 women, and of these, 9 were smokers, and 3 scored ≥ 8 points in the AUDIT. The sample data are described in Table 1. Regarding the tobacco use duration in the cancer group, it was observed that the men had used tobacco for the longest time compared to the women (40 ± 20 and 24 ± 22, years, median ± IQR). The score results are described in Table 2.

The Mann–Whitney test showed significant differences in the AUDIT score (*p* = 0.002) and *CYP1A1* expression (*p* = 0.015) between the cancer and control group. However, we found no differences in the number of cigarettes smoked per day (*p* = 0.256), duration of tobacco use (*p* = 0.059), or FTCD (*p* = 0.061). There were also no differences in the expression of *GSTM1* (*p* = 0.32), *GSTP1* (*p* = 0.398), or *GSTT1* (*p* = 0.133).

Participants in advanced stages showed significant differences in duration of tobacco use (*p* = 0.047), FTCD (*p* = 0.002), AUDIT score (*p* = 0.013), and *CYP1A1* expression (*p* = 0.002) when compared to patients in early stages. Nevertheless, no significant differences were observed in the number of cigarettes smoked per day (*p* = 0.135), nor in the expression of *GSTM1* (*p* = 0.336), *GSTP1* (*p* = 0.357), or *GSTT1* (*p* = 0.50).

Smokers had larger tumors (*p* = 0.012) and more regional metastases (*p* = 0.044) than non-smokers. We also found higher AUDIT scores among smokers (*p* < 0.001), even among those with cancer (*p* = 0.017), when compared to non-smokers.

Considering the whole sample, Spearman’s correlation coefficient showed significant correlations between tumor size and duration of tobacco use (*p* = 0.036), AUDIT score (*p* = 0.001), and *CYP1A1* expression (*p* = 0.002). The presence of regional metastases was correlated with higher AUDIT score (*p* = 0.009) and higher expression of *CYP1A1* (*p* = 0.014). We also observed significant correlations of the AUDIT score with the expression of *CYP1A1* (*p* = 0.002) and tobacco consumption parameters, such as number of cigarettes smoked per day (*p* = 0.033) and duration of tobacco use (*p* = 0.002) (Table 3).

In the group of patients with early-stage cancer, we found strong correlations between the AUDIT score and the number of cigarettes smoked per day (*p* = 0.010), duration of tobacco use (*p* = 0.005), and FTCD (*p* = 0.001) (Table 4). Among patients in advanced stages, there was a significant correlation between regional metastases and the number of cigarettes smoked per day (*p* = 0.046).

Among the subjects in the control group, we observed a significant correlation between the age of the participants and the expression of *GSTP1* (*p* = 0.002). In addition, *GSTP1* expression was significantly correlated with the number of cigarettes smoked per day (*p* = 0.028), duration of tobacco use (*p* = 0.037), and FTCD (*p* = 0.041) (Table 5).

## 4. Discussion

Oral SCC is one of the most common malignancies worldwide, with higher prevalence in some regions. This cancer is responsible for high morbidity and mortality, usually because it is diagnosed in more advanced stages [32]. Multicenter studies conducted by large research consortia such as Gencapo [33], Arcage [34], Inhance [35], InterCHANGE [36], and, more recently, HeadSpace [37], mapped biomolecular characteristics of head and neck cancers, including oral cancer, in different populations. Although biomolecular science has made incredible advances for cancer study, unfortunately, its usefulness in daily medical care is available for few countries or institutions, becoming more used in research centers.

We chose to use internationally validated and accessible questionnaires to assess the degree of tobacco and alcohol dependence and compare it with the expression of genes involved in carcinogen metabolism [38]. In this way, those questionnaires could be a useful tool to estimate indirectly the degree of exposure to tobacco and alcohol harmful products, as well as the gene expression in patients with oral cancer [39].

Alcohol acts as a trigger of tobacco use and vice versa. In our series, all smokers with cancer were regular consumers of alcohol, with mainly high-risk consumption. We also observed that most of the heavy drinkers were also heavy smokers and more nicotine dependent. These findings illustrate the multiple chemical addictions (tobacco and alcohol) in patients with head and neck cancer [40]. This profile differs from the results reported by Graner et al. [41] for patients diagnosed with oral cancer, with only 32.6% being alcoholics. Our data showed that the majority of patients who reported no alcohol use were also non-smokers, even in the control group.

Another finding was that gender had an impact on the disease stage. Most cases in patients with advanced diagnosis were males, which corresponds to the Brazilian reality characterized by higher male prevalence and late diagnosis of the disease in men [42,43]. This is due to the fact that men usually have a higher tolerance for discomfort, postponing the medical evaluation by health professionals. In addition, men tend to neglect their symptoms, either for fear of the diagnosis or for issues related to the impossibility of working, dependence on family or friends, and feelings of inferiority [44].

The *CYP1A1* gene is one of the main genes responsible for the metabolism of alcohol and tobacco carcinogens [45]. The correlation between *CYP1A1* and some Phase II genes seems to be influenced by tissue factors themselves that change the synergy effects between the genes [46]. We did not observe differences in *GSTM1*, *GSTP1*, or *GSTT1* gene expression in the cancer group, but as Phase II enzymes are responsible for detoxification of carcinogens, once cancer is present, it possibly means that the role of these enzymes is not proceeded to neutralize the toxic substances. The greater correlation found between *CYP1A1* expression and tumor size, and regional metastases reinforce the hypothesis that the more bioactivation and presence of carcinogens, the poorest outcome can be found [47].

Since Phase I and II enzymes show an opposite reaction regarding activation and detoxification of the carcinogens, it was expected that *CYP1A1* expression would be higher in cancer samples. On the other hand, *GSTP1* was predominant among benign lesions and significantly correlated smoking parameters such as number of cigarettes smoked per day, duration of tobacco consumption, and nicotine dependence assessed by TFCD, providing a probable protective action to smokers. The *CYP1A1* and *GTSP1* results found in our study may indicate that this hypothesis may be true.

This correlation between *CYP1A1*, *GSTM1*, *GSTP1*, and *GSTT1* seems to be important, once this balance between activation and detoxification of carcinogens can influence the development, progression, and prevention of cancer. This relationship was found with the polymorphic forms and has been associated with other types of malignancies such as breast, colon, and lung cancer. Besides that, the research of polymorphic genes in head and neck cancer is more prevalent in specific countries such as India, China, Japan, Spain, and Germany. Unfortunately, Brazil did not have scientific data regarding the prevalence of these polymorphisms and due to our population characteristics, we decided not to include polymorphic forms of those genes in our study [48,49,50,51,52].

The *CYP1A1* gene was significantly correlated with the instruments chosen to estimate the parameters of tobacco and alcohol consumption (FTCD and AUDIT scores), confirming the already known statement that the dual use of these substances substantially increases the risk of developing cancer. To the best of our knowledge, this correlation has not been evaluated before in Brazil. Our results partially disagree with the findings of Masood et al. [46] who observed reduced expression of *CYP1A1* in some patients with oral cancer, but higher expression in cases in advanced stages. In our series, the number of cigarettes smoked per day and the exposure time were determinant for the tumor stage, represented by the variable tumor size and regional metastasis.

Older patients with a higher history of tobacco consumption and with benign lesions expressed more GSTs, indicating that this gene should be an important protective factor in this group of patients. Could *GSTP1* thus be considered a marker? It appears that, even though cancer development is multifactorial, through the study of gene expression in this scenario, we can draw some conclusions. It was possible to demonstrate that the gene expression of some carcinogen metabolizers can vary depending on the degree of exposure of individuals to agents such as tobacco and alcohol. The mutual analysis of the Fagerström and AUDIT questionnaires combined with the gene expression study was superior to the single gene expression analysis to determine the risk of oral cancer. Thus, the clinical aspects obtained by the questionnaires must be valued, and not just the molecular factors.

These questionnaires are easy to apply and can be used by any healthcare professional. Few studies have applied these tools in the context of oncology. In a recent study, Yokoyama et al. [53] used the AUDIT for the follow-up of patients with esophageal cancer, showing its clinical applicability in the early diagnosis of a second primary tumor. The same approach can be used to follow-up on oral cancer patients, encouraging them to stop their harmful habits and promoting better health.

The assessment of nicotine dependence is still not a routine procedure in initial oncological assessment, although continued smoking after a cancer diagnosis is associated with a poor prognosis. Almeida et al. [54] described high nicotine dependence among patients with head and neck cancer and highlighted the need to assess the smoking profile of cancer patients. In another study, Schiller et al. [55] reported some aspects of the profile of patients with head and neck cancer related to tobacco and alcohol consumption using the same instruments. The authors also emphasized the issue of multiple addictions and the lack of smoking and alcohol cessation programs in this specific group of patients in cancer treatment services.

As a limiting factor of our work, we must emphasize that it was not possible to carry out a complementary immunohistochemical analysis for all samples.

## 5. Conclusions

Motivating health professionals, especially oncologists, to evaluate smokers’ nicotine dependence and encouraging them to participate in cessation programs are important educational measures and can undoubtedly impact the outcomes not only for disease prevention, whether malignant or not, related to tobacco and alcohol dependence, but also to the treatment response. The FTCD and AUDIT questionnaires designed to evaluate and estimate the degree of exposure and dependence to tobacco and alcohol combined with simple gene expression tests can be useful tools to assess the risks of developing oral cancer.

## Figures and Tables

**Table 1:** Demographic data of the sample.

Sample	*n* = 48			
Age (Years, Mean ± SD)	55 ± 14.5			
	Cancer group		Control group	
	*n* = 33 (75)		*n* = 15 (25)	
Gender	Male	Female	Male	Female
Subjects (*n*, %)	24 (50)	9 (18.75)	8 (3.84)	7 (3.36)
Cancer stage				
Stage I	2	-	-	-
Stage II	3	6	-	-
Stage III	4	1	-	-
Stage IV	15	2	-	-
AUDIT score (median ± IQR)	12 ± 6.5	4 ± 5	*	
≥8 points	19	2	2	1
<8 points	5	7	6	6

SD: standard deviation, IQR: interquartile range. * Statistical analysis not performed due to sample size.

**Table 2:** Description of FTCD and AUDIT score results.

FTCD Score of Dependence	Cancer Group (*n*/%)	Control Group (*n*/%)
0–2 = very low dependence	3 (8.33)	2 (5.56)
3–4 = low dependence	6 (16.66)	1 (2.78)
5 = moderate dependence	4 (11.11)	3 (8.33)
6–7 = high dependence	11 (30.555)	2 (5.56)
8–10 = very high dependence	3 (8.33)	1 (2.78)
Total	27	9
AUDIT Score		
0–7 points = low risk use	10 (26.32)	4 (10.53)
8–15 = risk use	14 (3.84)	2 (5.26)
16–19 points = harmful use	5 (13.16)	1 (2.63)
≥20 points = probable dependence	2 (5.26)	-
Total	31 *	7

* 2 subjects with cancer declared not using tobacco and alcohol.

**Table 3:** Spearman’s correlation coefficient of whole sample.

		Cigarettes Per Day	Duration of Tobacco Use	Tumor Size	Nodal Metastasis	AUDIT Score	*CYP1A1*
Cigarettes per day	Rho						
	*p*-value						
Duration of tobacco use	Rho	0.606					
	*p*-value	0.000					
Tumor size	Rho	0.143	0.304				
	*p*-value	0.333	0.036				
Nodal metastasis	Rho	0.249	0.073	0.536			
	*p*-value	0.088	0.624	0.000			
AUDIT score	Rho	0.308	0.433	0.483	0.371		
	*p*-value	0.033	0.002	0.001	0.009		
*CYP1A1*	Rho	0.057	0.285	0.361	0.353	0.445	
	*p*-value	0.702	0.050	0.002	0.014	0.002	

Rho: Spearman’s Rho.

**Table 4:** Spearman’s correlation coefficient of early-stage cancer.

		Cigarettes Per Day	Duration of Tobacco Use	Fagerström Test	AUDIT Score
Cigarettes per day	Rho				
	*p*-value				
Duration of tobacco use	Rho	0.861			
	*p*-value	0.003			
Fagerström test	Rho	0.701	0.784		
	*p*-value	0.019	0.007		
AUDIT score	Rho	0.761	0.800	0.876	
	*p*-value	0.010	0.005	0.001	

Rho: Spearman’s Rho.

**Table 5:** Spearman’s correlation coefficient of the control group.

		Age	Smoking Status	Cigarettes per Day	Duration of Tobacco Use	Fagerström Test	*GSTP1*
Age	Rho						
	*p*-value						
Smoking status	Rho	0.506					
	*p*-value	0.066					
Cigarettes per day	Rho	0.597	0.880				
	*p*-value	0.021	0.000				
Duration of tobacco use	Rho	0.511	0.887	0.812			
	*p*-value	0.003	0.000	0.000			
Fagerström test	Rho	0.491	0.882	0.987	0.839		
	*p*-value	0.066	0.000	0.000	0.000		
*GSTP1*	Rho	0.730	0.601	0.566	0.542	0.533	
	*p*-value	0.002	0.018	0.028	0.037	0.041	

Rho: Spearman’s Rho.

## Data Availability

Not applicable.

## References

[1] Cancer. https://www.who.int/news-room/fact-sheets/detail/cancer.

[2] Wünsch Filho V., Mirra A.P., López R.V.M., Antunes L.F. (2010). Tabagismo e câncer no Brasil: Evidências e perspectivas. Rev. Bras. Epidemiol..

[3] Curado M.P., Hashibe M. (2009). Recent changes in the epidemiology of head and neck cancer. Curr. Opin. Oncol..

[4] Barnes L., Eveson J., Reichart P., Sidransky D., WHO (2005). World Health Organization Classification of Tumours. Pathology and Genetics of Head and Neck Tumours.

[5] Huang S.H., O’Sullivan B. (2017). Overview of the 8th Edition TNM Classification for Head and Neck Cancer. Curr. Treat. Options Oncol..

[6] Glynn T., Seffrin J.R., Brawley O.W., Grey N., Ross H. (2010). The globalization of tobacco use: 21 challenges for the 21st century. CA Cancer J. Clin..

[7] World Health Organization—WHO (2016). ICD-10 Version: 2016. https://icd.who.int/browse10/2016/en#/.

[8] Warnakulasuriya S., Dietrich T., Bornstein M.M., Peidró E.C., Preshaw P.M., Walter C., Wennström J.L., Bergström J. (2010). Oral health risks of tobacco use and effects of cessation. Int. Dent. J..

[9] Winn D.M., Lee Y.A., Hashibe M., Boffetta P., The INHANCE Consortium (2015). The INHANCE consortium: Toward a better understanding of the causes and mechanisms of head and neck cancer. Oral Dis..

[10] Hashibe M., Brennan P., Chuang S.C., Boccia S., Castellsague X., Chen C., Curado M.P., Dal Maso L., Daudt A.W., Fabianova E. (2009). Interaction between tobacco and alcohol use and the risk of head and neck cancer: Pooled analysis in the INHANCE consortium. Cancer Epidemiol. Prev. Biomark..

[11] Szymańska K., Hung R.J., Wünsch-Filho V., Eluf-Neto J., Curado M.P., Koifman S., Matos E., Menezes A., Fernandez L., Daudt A.W. (2011). Alcohol and tobacco, and the risk of cancers of the upper aerodigestive tract in Latin America: A case-control study. Cancer Causes Control.

[12] Dobrossy L. (2005). Epidemiology of head and neck cancer: Magnitude of the problem. Cancer Metastasis Rev..

[13] Gandini S., Botteri E., Iodice S., Boniol M., Lowenfels A.B., Maisonneuve P., Boyle P. (2008). Tobacco smoking and cancer: A meta-analysis. Int. J. Cancer.

[14] Petti S. (2009). Lifestyle risk factors for oral cancer. Oral Oncol..

[15] Marron M., Boffetta P., Zhang Z.-F., Zaridze D., Wünsch-Filho V., Winn D.M., Wei Q., Talamini R., Szeszenia-Dabrowska N., Sturgis E.M. (2010). Cessation of alcohol drinking, tobacco smoking and the reversal of head and neck cancer risk. Int. J. Epidemiol..

[16] Rendic S.P., Guengerich F.P. (2021). Human Family 1–4 Cytochrome P450 Enzymes Involved in the Metabolic Activation of Xenobiotic and Physiological Chemicals: An Update. Arch. Toxicol..

[17] Varela-Lema L., Taioli E., Ruano-Ravina A., Barros-Dios J.M., Anantharaman D., Benhamou S., Boccia S., Bhisey R.A., Cadoni G., Capoluongo E. (2008). Meta-Analysis and Pooled Analysis of GSTM1 and CYP1A1 Polymorphisms and Oral and Pharyngeal Cancers: A HuGE-GSEC Review. Genet. Med..

[18] Singh R.R., Reindl K.M. (2021). Glutathione S-Transferases in Cancer. Antioxidants.

[19] Bongers V., Snow G.B., Braakhuis B.J.M. (1995). The role of glutathione S-transferases in head and neck squamous cell carcinogenesis. Eur. J. Cancer Part B Oral Oncol..

[20] Stewart B.W., Wild C., World Health Organization (2014). World Cancer Report 2014.

[21] Gattás G.J.F., De Carvalho M.B., Siraque M.S., Curioni O.A., Kohler P., Eluf-Neto J., Wünsch-Filho V. (2006). Genetic polymorphisms of CYP1A1, CYP2E1, GSTM1, and GSTT1 associated with head and neck cancer. Head Neck..

[22] De Amorim L.M.D.F., Rossini A., Mendonça G.A.S., Lotsch P.F., de Almeida Simão T., de Moura Gallo C.V., Pinto L.F.R. (2002). CYP1A1, GSTM1, and GSTT1 polymorphisms and breast cancer risk in Brazilian women. Cancer Lett..

[23] Rossini A., Rapozo D., Lima S.S., Guimaraes D., Ferreira M., Teixeira R., Kruel C., Barros S., Andreollo N., Acatauassu R. (2007). Polymorphisms of GSTP1 and GSTT1, but not of CYP2A6, CYP2E1 or GSTM1, modify the risk for esophageal cancer in a western population. Carcinogenesis.

[24] Rossini A., Rapozo D.C.M., Amorim L., Macedo J.M.B., Medina R., Neto J.F.N., Gallo C.V.M., Pinto L.F.R. (2002). Frequencies of GSTM1, GSTT1, and GSTP1 polymorphisms in a Brazilian population. Genet. Mol. Res..

[25] Pinto L.F.R., Rossini A.M.T., Albano R.M., Felzenszwalb I., de Moura Gallo C.V., Nunes R.A., Andreollo N.A. (2003). Mechanisms of esophageal cancer development in Brazilians. Mutat Res..

[26] Heatherton T.F., Kozlowski L.T., Frecker R.C., Fagerström K.O. (1991). The Fagerström Test for Nicotine Dependence: A revision of the Fagerström Tolerance Questionnaire. Br. J. Addict..

[27] Moretti-Pires R.O., Corradi-Webster C.M. (2011). Adaptation and validation of the Alcohol Use Disorders Identification Test (AUDIT) for a river population in the Brazilian Amazon. Cad. Saude Publica.

[28] Amin M.B., Greene F.L., Edge S.B., Compton C.C., Gershenwald J.E., Brookland R.K., Meyer L., Gress D.M., Byrd D.R., Winchester D.P. (2017). The Eighth Edition AJCC Cancer Staging Manual: Continuing to build a bridge from a population-based to a more “personalized” approach to cancer staging. CA. Cancer J. Clin..

[29] Fagerström K. (2012). Determinants of tobacco use and renaming the FTND to the Fagerström Test for Cigarette Dependence. Nicotine Tob. Res..

[30] Huang C.L., Lin H.H., Wang H.H. (2008). Evaluating screening performances of the Fagerström tolerance questionnaire, the Fagerstrom test for nicotine dependence and the heavy smoking index among Taiwanese male smokers. J. Clin. Nurs..

[31] Livak K.J., Schmittgen T.D. (2001). Analysis of relative gene expression data using real-time quantitative PCR and the 2(-Delta Delta C(T)) Method. Methods.

[32] Bandeira C.M., de Almeida A.Á., Carta C.F.L., Almeida J.D., Kaminagakura E. (2017). How to improve the early diagnosis of oral cancer?. Braz. Dent. Sci..

[33] GENCAPO. http://www.gencapo.famerp.br/lmmb/.

[34] Canova C., Hashibe M., Simonato L., Nelis M., Metspalu A., Lagiou P., Trichopoulos D., Ahrens W., Pigeot I., Merletti F. (2009). Genetic Associations of 115 Polymorphisms with Cancers of the Upper Aerodigestive Tract across 10 European Countries: The ARCAGE Project. Cancer Res..

[35] International Head and Neck Cancer Epidemiology Consortium|University of Utah. https://medicine.utah.edu/inhance/.

[36] IARCRP: Home. https://interchange.iarc.fr/.

[37] HEAD Space: Home. https://headspace.iarc.fr/.

[38] Di Credico G., Polesel J., Dal Maso L., Pauli F., Torelli N., Luce D., Radoï L., Matsuo K., Serraino D., Brennan P. (2020). Alcohol drinking and head and neck cancer risk: The joint effect of intensity and duration. Br. J. Cancer.

[39] Masood N., Malik F.A., Kayani M.A. (2011). Expression of xenobiotic metabolizing genes in head and neck cancer tissues. Asian Pac. J. Cancer Prev..

[40] Aupérin A. (2020). Epidemiology of head and neck cancers: An update. Curr. Opin. Oncol..

[41] Graner K.M., Rolim G.S., Moraes A.B.A., Padovani C.R., Lopes M.A., Santos-Silva A.R., A Ramos-Cerqueira A.T. (2016). Feelings, perceptions, and expectations of patients during the process of oral cancer diagnosis. Support Care Cancer.

[42] Ministério da Saúde (2019). Estimate/2020—Cancer Incidence in Brazil.

[43] Lipsky M.S., Su S., Crespo C.J., Hung M. (2021). Men and Oral Health: A Review of Sex and Gender Differences. Am. J. Men’s Health.

[44] Smith L.K., Pope C., Botha J.L. (2005). Patients’ help-seeking experiences and delay in cancer presentation: A qualitative synthesis. Lancet.

[45] Rendic S. (2002). Summary of information on human CYP enzymes: Human P450 metabolism data. Drug Metab. Rev..

[46] Masood N., Kayani M.A. (2012). Expression patterns of carcinogen detoxifying genes (CYP1A1, GSTP1 & GSTT1) in HNC patients. Pathol. Oncol. Res..

[47] Zakiullah Z., Ahmadullah A., Khisroon M., Saeed M., Khan A., Khuda F., Ali S., Javed N., Ovais M., Masood N. (2015). Genetic susceptibility to oral cancer due to combined effects of GSTT1, GSTM1 and CYP1A1 gene variants in tobacco addicted patients of pashtun ethnicity of Khyber Pakhtunkhwa Province of Pakistan. Asian Pac. J. Cancer Prev..

[48] Losi-Guembarovski R., Cólus I.M., de Menezes R.P., Poliseli F., Chaves V.N., Kuasne H., Leichsenring A., Guembarovski A.L., Oliveira B.W., Ramos G. (2008). Lack of association among polymorphic xenobiotic-metabolizing enzyme genotypes and the occurrence and progression of oral carcinoma in a Brazilian population. Anticancer. Res..

[49] Singh R., Haridas N., Shah F., Patel J., Shukla S., Patel P. (2014). Gene Polymorphisms, Tobacco Exposure and Oral Cancer Susceptibility: A Study from GUJARAT, West India. Oral Dis..

[50] Androutsopoulos V.P., Tsatsakis A.M., Spandidos D.A. (2009). Cytochrome P450 CYP1A1: Wider Roles in Cancer Progression and Prevention. BMC Cancer.

[51] Chatterjee S., Chakrabarti S., Sengupta B., Poddar S., Biswas D., Sengupta S., Talukder G. (2009). Prevalence of CYP1A1 and GST Polymorphisms in the Population of Northeastern India and Susceptibility of Oral Cancer. Oncol. Res..

[52] He X., Feng S. (2015). Role of Metabolic Enzymes P450 (CYP) on Activating Procarcinogen and Their Polymorphisms on the Risk of Cancers. Curr. Drug Metab..

[53] Yokoyama A., Katada C., Yokoyama T., Takizawa K., Yano T., Oda I., Shimizu Y., Nakanishi H., Koike T., Hirao M. (2020). The Alcohol Use Disorders Identification Test and the risk of metachronous cancer after endoscopic resection of esophageal cancer. Carcinogenesis.

[54] Almeida A.A., Bandeira C.M., Goncalves A.J., Araujo A.J. (2014). Nicotine dependence and smoking habits in patients with head and neck cancer. J. Bras. Pneumol..

[55] Schiller U., Inhestern J., Burger U., Singer S., Guntinas-Lichius O. (2016). Predictors of post-treatment smoking and drinking behavior of head and neck cancer survivors: Results of a population-based survey. Eur. Arch. Oto-Rhino-Laryngol..

